# Supplementary material for: A secreted microRNA disrupts autophagy in distinct tissues of Caenorhabditis elegans upon ageing
Source: Nat Commun. 2019 Oct 23;10:4827. doi: 10.1038/s41467-019-12821-2 (PMC6811558; doi:10.1038/s41467-019-12821-2)
Supplement: Supplementary file 1 — Supplementary Information [file 41467_2019_12821_MOESM1_ESM.pdf]

Supplementary Information

A secreted microRNA disrupts autophagy in distinct tissues upon ageing

Yifei Zhou[1], Xueqing Wang[1], Mengjiao Song[1], Zhidong He[1], Guizhong Cui[1], Guangdun Peng[2, 3], Naihe Jing[1] and Yidong Shen[1*]

Affiliations:

[1] State Key Laboratory of Cell Biology, Innovation Center for Cell Signaling Network, CAS Center for Excellence in Molecular Cell Science, Shanghai Institute of Biochemistry and Cell Biology, University of Chinese Academy of Sciences，Chinese Academy of Sciences

320 Yueyang Rd.

200031 Shanghai, China

[2] CAS Key Laboratory of Regenerative Biology, Guangdong Provincial Key Laboratory of Stem Cell and Regenerative Medicine, Guangzhou Institutes of Biomedicine and Health, Chinese Academy of Sciences

510530 Guangzhou, China

[3] Guangzhou Regenerative Medicine and Health Guangdong Laboratory (GRMH-GDL)

510005 Guangzhou, China

*Correspondence to: yidong.shen@sibcb.ac.cn.

Tel: +86-21-54921171

Supplementary Figures

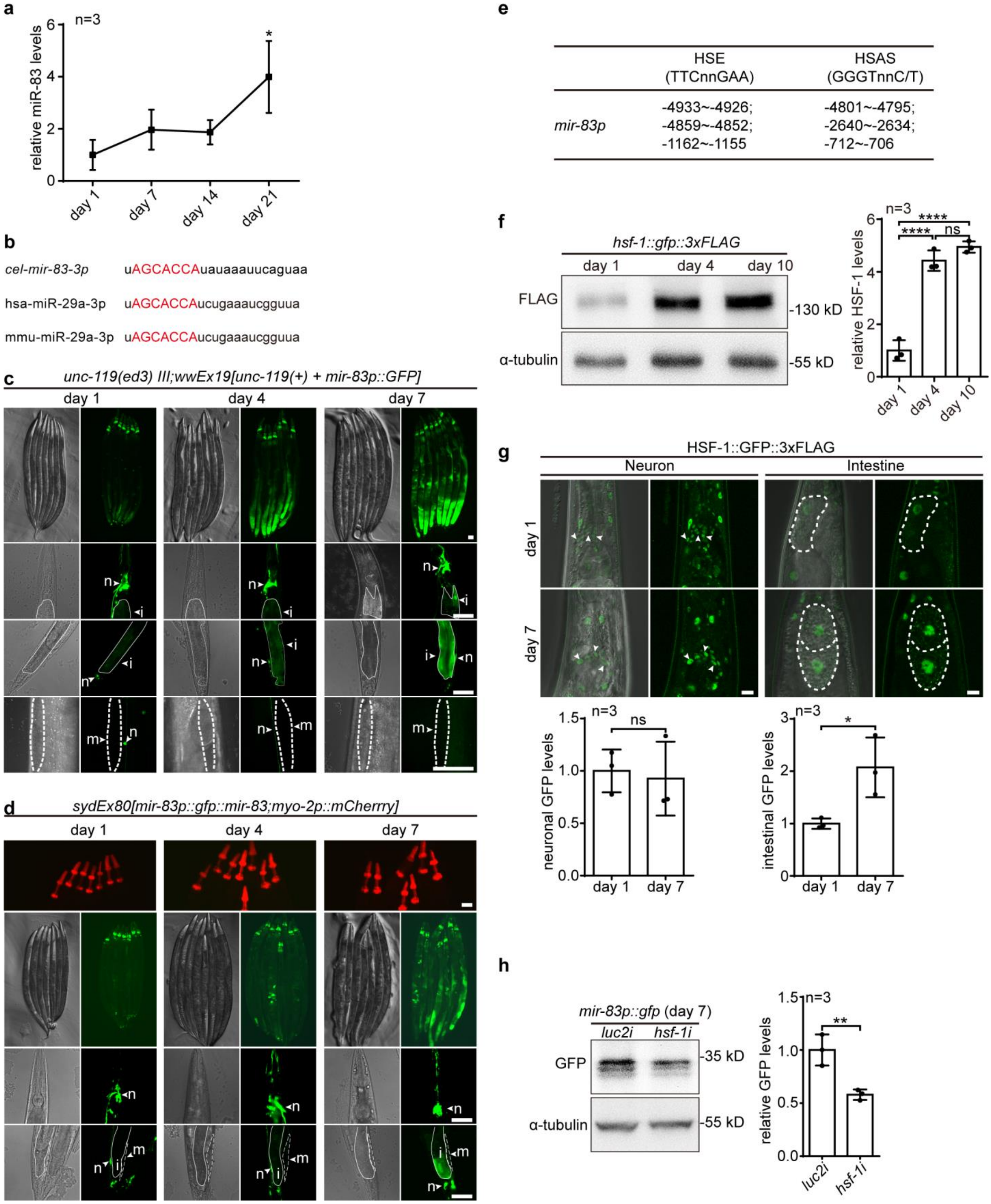

Supplementary Fig. 1. *mir-83* is upregulated by *hsf-1* with ageing.

a, RNA-Seq results of *mir-83* in the WT worms of indicated ages. The data were normalized against the miR-83 level at day 1. n=3 independent experiments.

b, Sequences of *cel-mir-83*, hsa-miR-29a and mmu-miR-29a. Uppercase letters in red denote seed sequences.

c-d, Representative images of two transcriptional transgenes of *mir-83* at indicated ages. GFP signals were observed in neurons and intestines (solid lines), but not detected in the body wall muscle (dashed lines). n: neuron, i: intestine, m: body wall muscle. Scale bars: 50 μm.

e, Heat shock elements (HSEs) and heat shock associated sites (HSASs) in the 5-kb promoter of *mir-83*.

f, The protein level of HSF-1 increases with ageing. α-tubulin and day 1 blots serve as controls for loading and normalization respectively. n=3 independent experiments.

g, HSF-1 is upregulated in the intestine but not in the head neurons of aged worms. Scale bar: 10 μm. Signals from day 1 worms serve as controls for normalization. n=3 independent experiments containing at least 22 worms.

h, Immunoblots of *mir-83p::*GFP treated with *luc2* RNAi or *hsf-1* RNAi at day 7. RNAi treatment was preformed from hatching to day 7 of adulthood. α-tubulin and worms under *luc2* RNAi serve as controls for loading and normalization respectively. n=3 independent experiments.

Statistical significance was calculated by one-way ANOVA in **a** and **f** or unpaired *t*-test in **g** and **h**. ns: non-significant, * p<0.05, ** p<0.01, **** p<0.0001. Source data

are provided as a Source Data file.

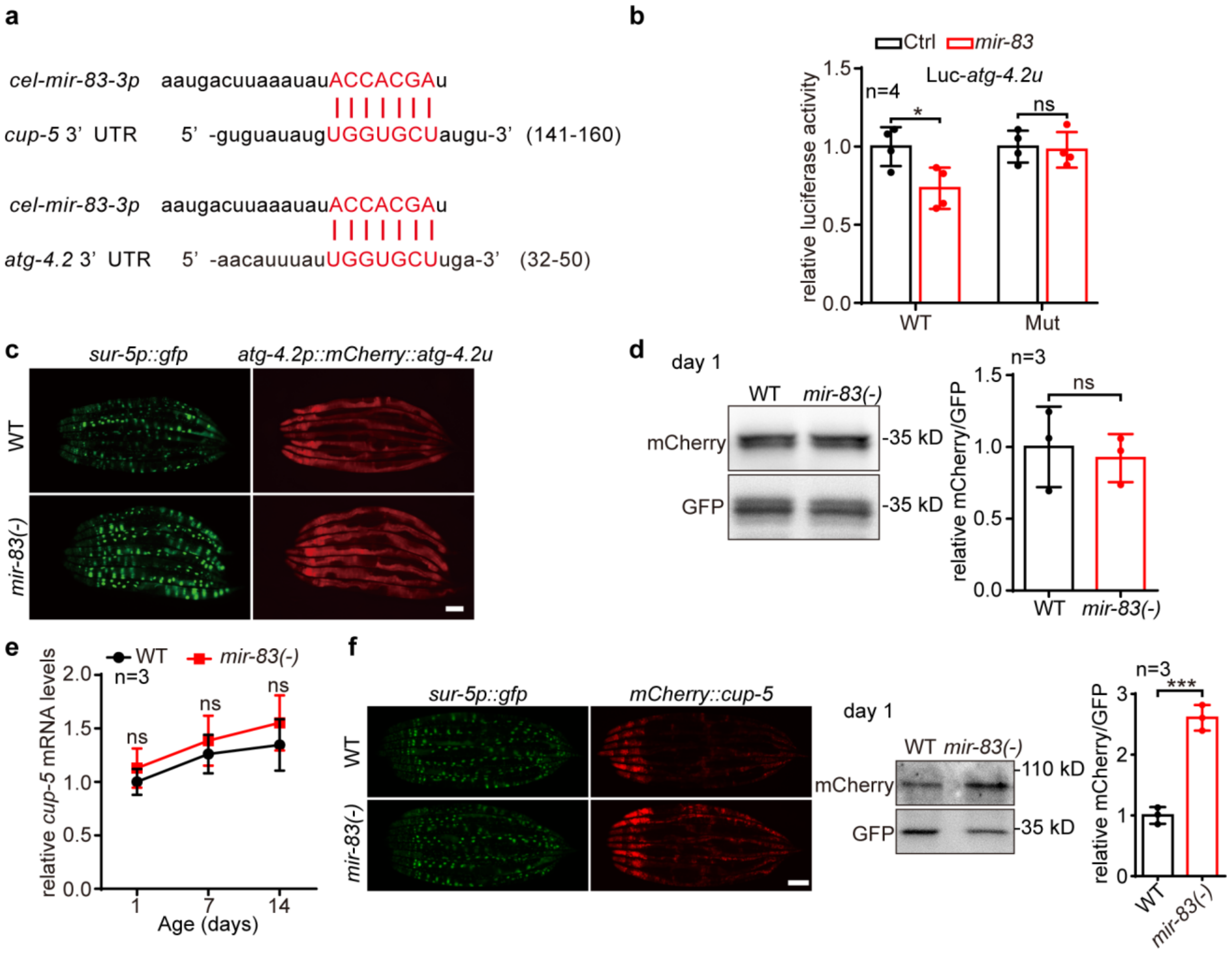

Supplementary Fig. 2. ***mir-83*** **targets** ***cup-5*** **but not** ***atg-4.2 in vivo*****.**

a, Predicted binding sites of *mir-83* on the 3'-UTRs of *cup-5* and *atg-4.2*.

b, The transfection of *cel-mir-83-3p* mimic suppresses a luciferase reporter with *atg-4.2* 3'-UTR in HEK293T cells. Ctrl transfected cells serve as controls for normalization. Mutating the *mir-83* binding site in *atg-4.2* 3'-UTR (Mut) blocked this interaction. n=4 independent experiments.

c-d, Fluorescent signals in **c,** and immunoblots in **d**, of the dual fluorescence reporter of *atg-4.2* 3'-UTR in the indicated strains at day 1 of adulthood. Blots against GFP

serve as a loading control. WT blots serve as the control for normalization. Scale bar: 100 μm. n=3 independent experiments.

e, *cup-5* mRNA levels in the WT worms and *mir-83(-)* mutants at indicated ages. Data were normalized against the expression of *cup-5* in the WT worms as day 1. n=3 independent experiments.

f, The protein level of mCherry::CUP-5 is increased upon mutating *mir-83,* whereas the expression of GFP remains unchanged in *mir-83(-)* mutants at day 1 of adulthood. WT blots serve as the control for normalization. n=3 independent experiments.

Statistical significance was calculated by unpaired *t*-test in **b**, **d**, and **f**, or two-way ANOVA in **e**. ns: non-significant, * $p<0.05$, *** $p<0.001$. Source data are provided as a Source Data file.

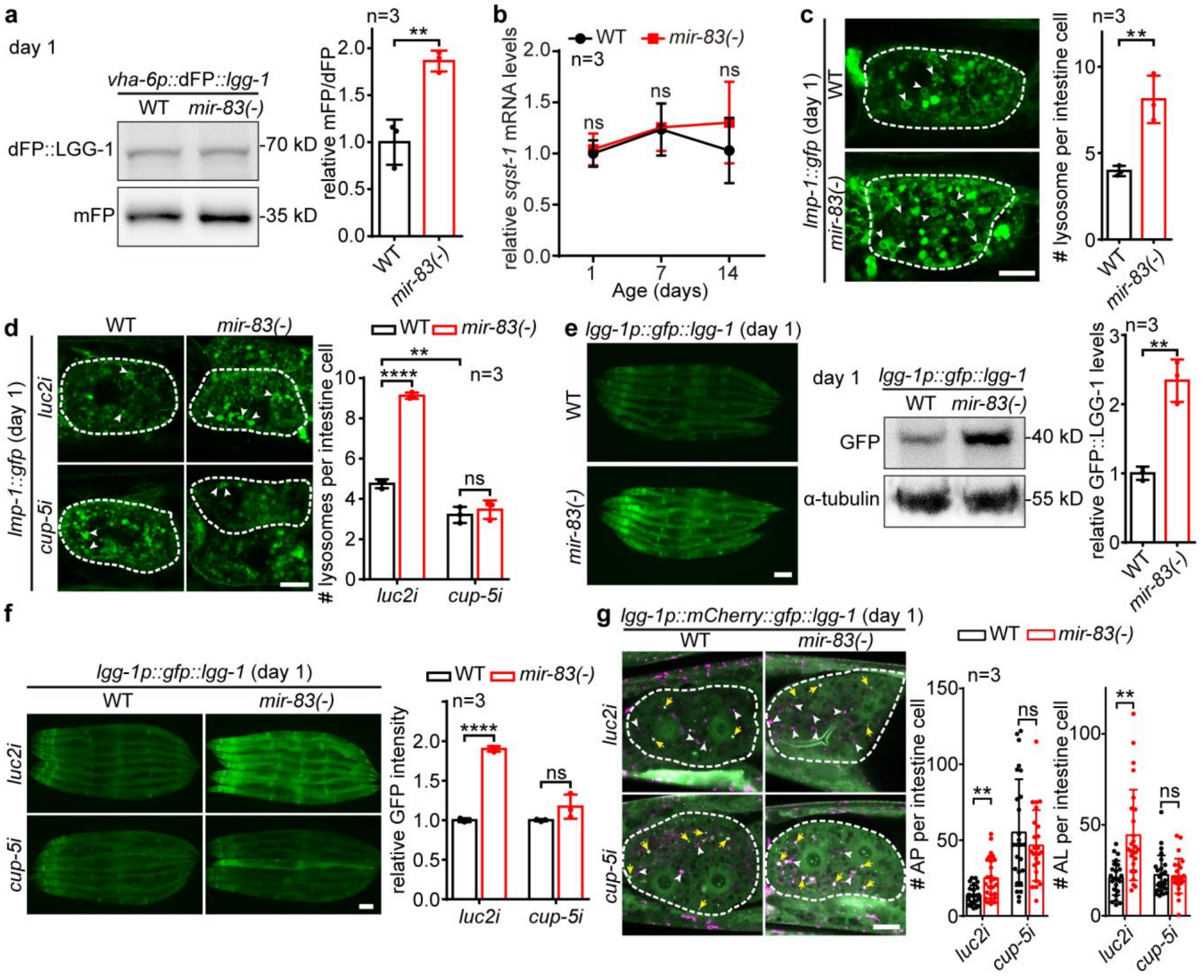

Supplementary Fig. 3. *mir-83* controls intestinal autophagy through regulating lysosome and LGG-1.

a, Immunoblots of the *vha-6p::dFP::lgg-1* reporter (mFP/dFP, mFP vs dFP::LGG-1) in indicated strains at day 1 of adulthood. WT blots serve as the control for normalization. n=3 independent experiments.

b, *sqst-1* mRNA levels in the WT worms and *mir-83(-)* mutants at indicated ages. Data were normalized against the *sqst-1* expression in the WT worms at day 1. n=3 independent experiments.

c, Lysosomes labelled by LMP-1-GFP (arrow heads) in the proximal intestine (dashed

lines) of the indicated strains at day 1 of adulthood. Scale bar: 10 μm. n=3 independent experiments containing at least 47 worms.

d, *cup-5* RNAi abolishes the increased lysosomes (LMP-1-GFP positive vacuoles, arrow heads) in the intestine of *mir-83(-)* mutants. Dashed lines denote intestinal cells. Scale bar: 10 μm. n=3 independent experiments containing at least 32 worms.

e, The expression of GFP::LGG-1 is increased in *mir-83(-)* mutants at day 1 of adulthood. Blots against α-tubulin and WT samples serve as controls for loading and normalization respectively. Scale bar: 100 μm. n=3 independent experiments.

f, *cup-5* RNAi abolishes the increased GFP::LGG-1 in *mir-83(-)* mutants at day 1 of adulthood. WT worms treated with *luc2* RNAi serve as controls for normalization. Scale bar: 100 μm. n=3 independent experiments containing at least 45 worms.

g, RNAi against *cup-5* blocks the enhanced autophagy in the intestine of *mir-83(-)* mutants at day 1 of adulthood. Scale bar: 100 μm. n=3 independent experiments containing at least 23 worms.

Statistical significance was calculated by unpaired *t*-test in **a**, **c**, and **e**, Two-way ANOVA in **b**, **d**, and **f**, or Poisson regression in **g**. ns: non-significant, ** $p<0.01$, **** $p<0.0001$. Source data are provided as a Source Data file.

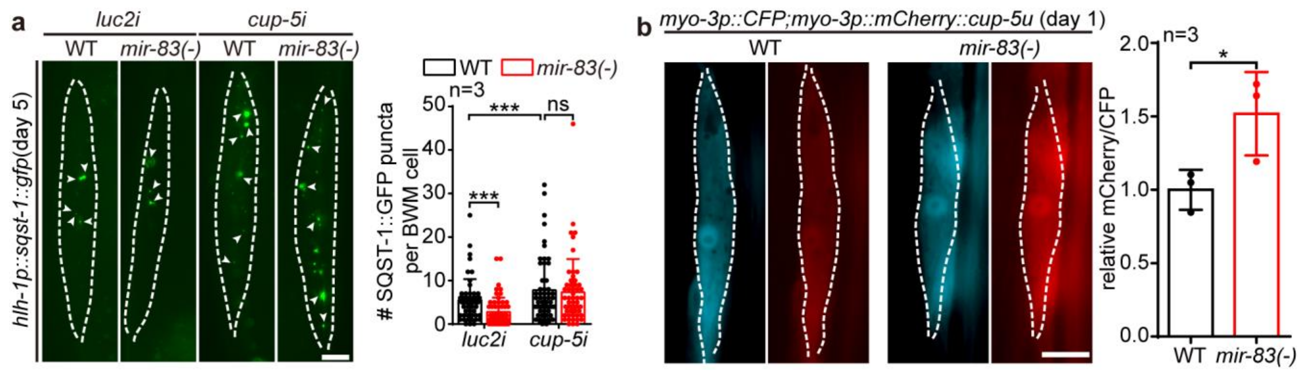

Supplementary Fig. 4. *mir-83* targets *cup-5* in the body wall muscle.

a, *cup-5* RNAi blocks the improved autophagy in the body wall muscle (BWM) of *mir-83(-)* mutants at day 5 of adulthood. n=3 independent experiments containing at least 52 worms.

b, Representative images of a BWM-specific dual fluorescence reporter of *cup-5* 3'-UTR in the WT worms and *mir-83(-)* mutants at day 1 of adulthood. Note that the BWM-specific mCherry signal controlled by *cup-5* 3'UTR is increased in *mir-83(-)* mutants. WT signals serve as the control for normalization. n=3 independent experiments containing at least 46 worms.

Dashed lines outline BWM cells. Scale bar: 10 µm. Statistical significance was calculated by Poisson regression in **a** or unpaired *t*-test in **b**. ns: non-significant, * $p<0.05$, *** $p<0.001$. Source data are provided as a Source Data file.

a

Tissue	Gene
Neuron	*rgef-1*
Intestine	*vha-6*
Body wall muscle	*myo-3*
Coelomocyte	*unc-122*
Hypodermis	*dpy-7*
Multiple tissues	*act-1*

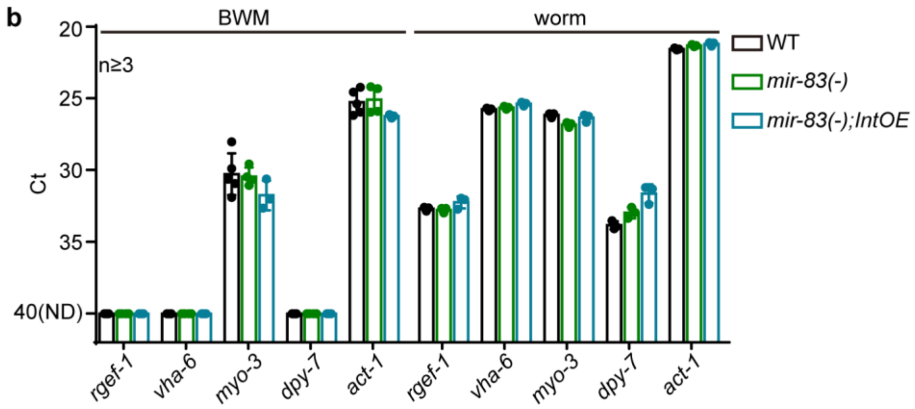

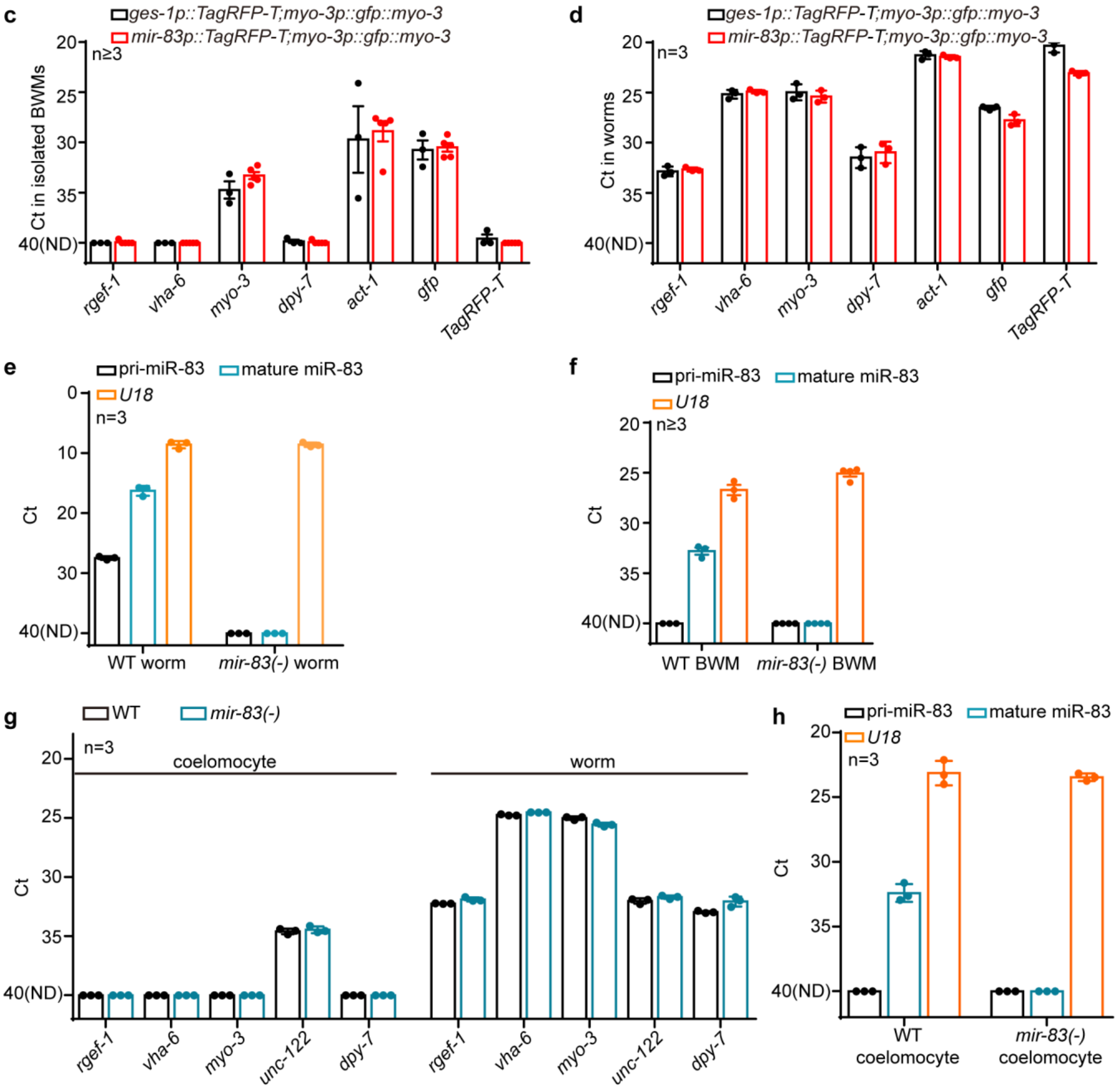

Supplementary Fig. 5. Intestinal *mir-83* is transferred into the body wall muscle.

a, A list of tissue-specific genes to examine the purity of isolated cells.

b, Quality control of isolated BWM cells from indicated strains by qRT-PCR. Note that the widely expressed *act-1* and the muscle-specific *myo-3* but no other tissue-specific genes are detected in the isolated BWM cells. *IntOE*: an intestine-specific transgene of *mir-83*. n=5, 4, and 3 independent experiments for WT, *mir-83(-),* and *mir-83(-);IntOE*, respectively.

c-d, qRT-PCR of indicated genes in the isolated BWM cells (**c**) or worms (**d**) of indicated strains. n=3 and 5 independent experiments for *ges-1p::TagRFP-T; myo-3p::gfp::myo-3* and *mir-83p::TagRFP-T; myo-3p::gfp::myo-3,* respectively in **c**. n=3 independent experiments in **d**.

e-f, qRT-PCR of the indicated forms of *mir-83* and U18 in worms (**e**), and isolated BWM cells (**f**). n=3 independent experiments in **e**. n=3 and 4 independent experiments for WT BWM and *mir-83(-)* BWM, respectively in **f**.

g, The isolated coelomocytes are free of contamination from the intestine or neurons. n=3 independent experiments.

h, The amount of different forms of *mir-83* in the isolated coelomocytes. n=3 independent experiments.

Ct values of samples without qRT-PCR signal were set as 40. ND: not detected. Source data are provided as a Source Data file.

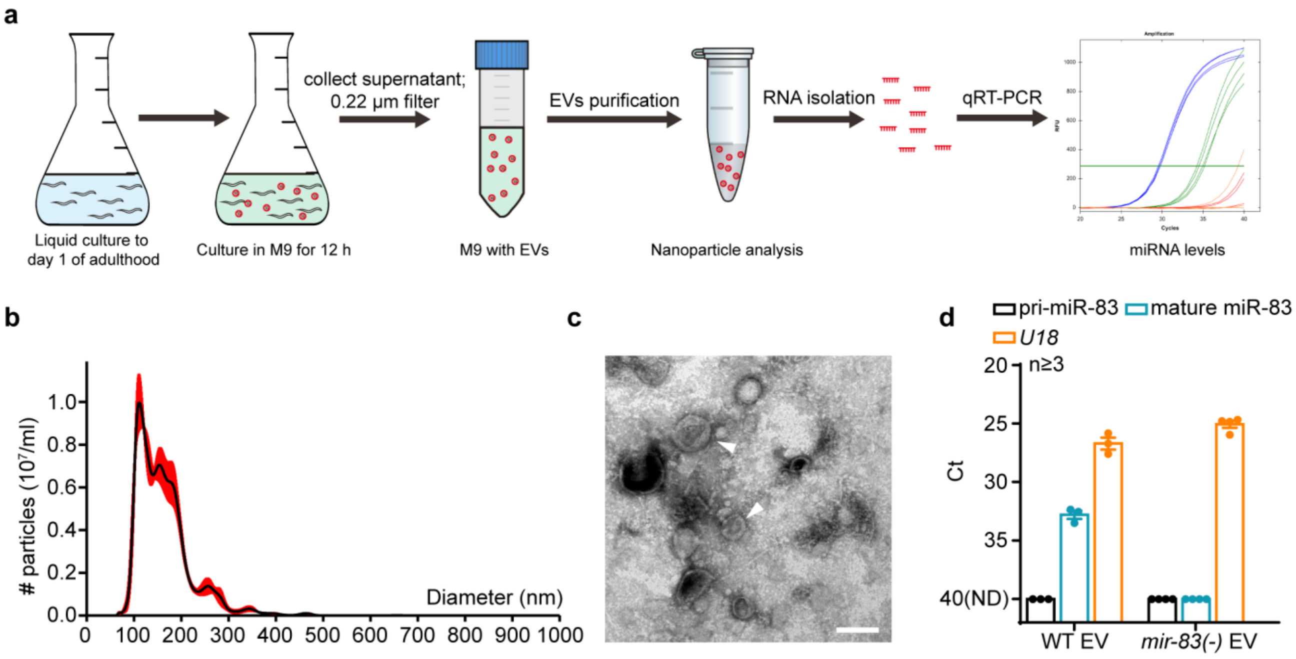

Supplementary Fig. 6. The collection of extracellular vesicles from worms.

a, The workflow to examine microRNA expression in the purified extracellular vesicles (EVs).

b, The size of purified EVs by nanoparticle analysis.

c, Electron microscopic examination of purified EVs (arrow heads). Scale bar: 100 nm.

d, The amount of different forms of *mir-83* in EVs. Ct values of samples without qRT-PCR signal were set as 40. ND: not detected. n=3 and 4 independent experiments for WT EV and *mir-83(-)* EV, respectively. Source data are provided as a Source Data file.

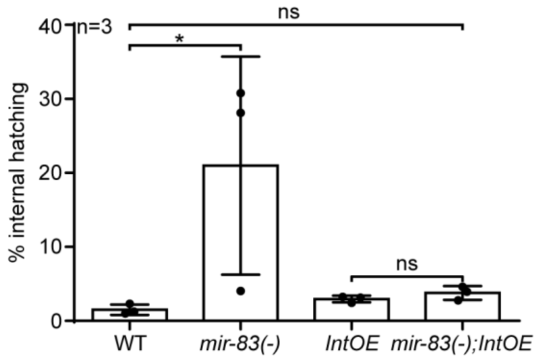

Supplementary Fig. 7. *mir-83* contributes to reproduction.

The internal hatching rate of indicated strains. Statistical significance was calculated by one-way ANOVA. ns: non-significant, * $p<0.05$. n=3 independent experiments containing total 360 worms. Source data are provided as a Source Data file.

Fig. 1b

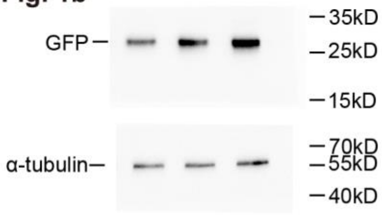

Fig. 2c

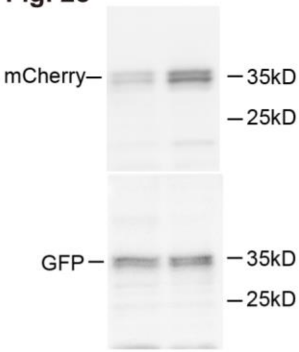

Fig. 2e

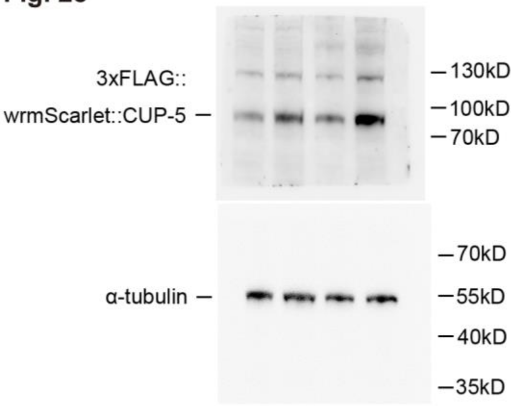

Fig. 3f

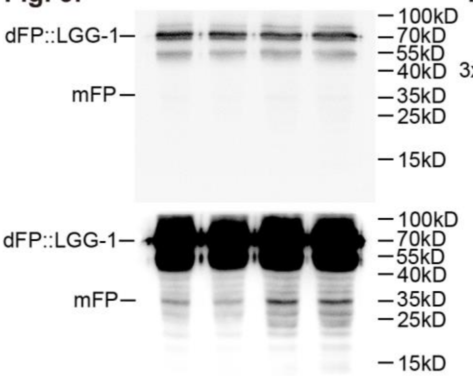

Fig. 5d

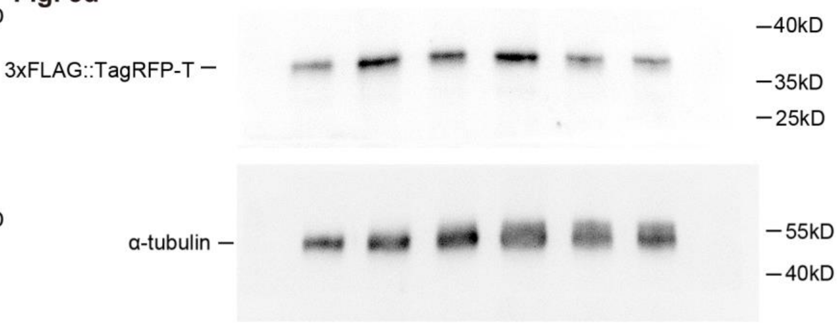

Supplementary Fig. 1f

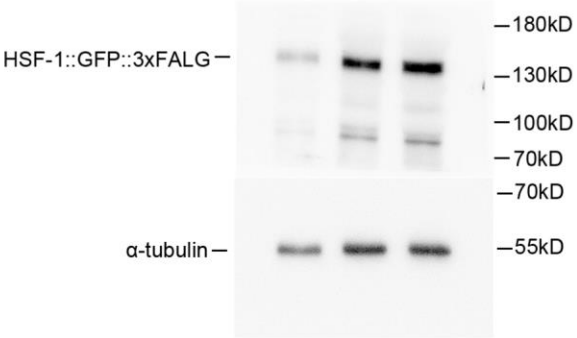

Supplementary Fig. 1h

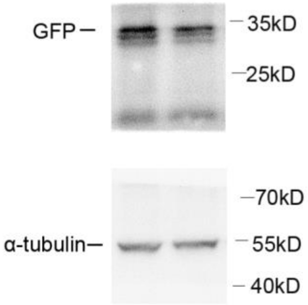

Supplementary Fig. 2d

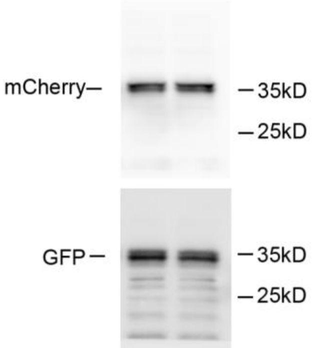

Supplementary Fig. 2f

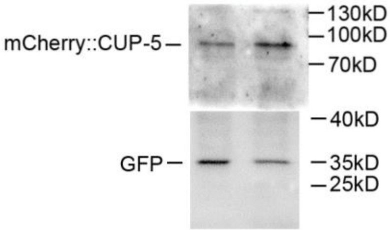

Supplementary Fig. 3a

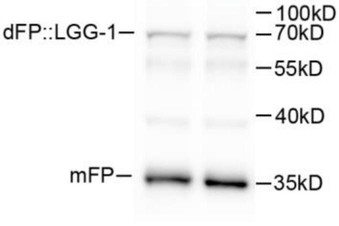

Supplementary Fig. 3e

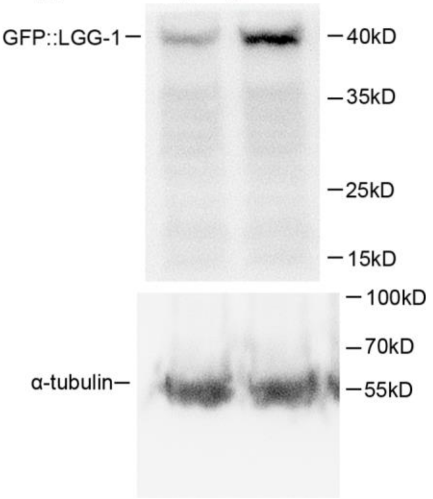

Supplementary Fig. 8. The uncropped and unprocessed images of western blot experiments shown in indicated figures.
