# Supplementary material for: A secreted microRNA disrupts autophagy in distinct tissues of Caenorhabditis elegans upon ageing
Source: Nat Commun. 2019 Oct 23;10:4827. doi: 10.1038/s41467-019-12821-2 (PMC6811558; doi:10.1038/s41467-019-12821-2)
Supplement: Supplementary file 7 — Supplementary Data descriptions [file 41467_2019_12821_MOESM7_ESM.pdf]

Description of Additional Supplementary Files

File Name: Supplementary Data 1.

Description: Lifespan analysis.

File Name: Supplementary Data 2.

Description: The statistics of all experiments except for lifespan analysis.

File Name: Supplementary Data 3.

Description: Strain list.

File Name: Supplementary Data 4.

Description: Oligo list.
